# Genetic variation in toll like receptors 2, 7, 9 and interleukin-6 is associated with cytomegalovirus infection in late pregnancy

**DOI:** 10.1186/s12881-020-01044-8

**Published:** 2020-05-25

**Authors:** Doreen Z. Mhandire, Kudakwashe Mhandire, Mulalo Magadze, Ambroise Wonkam, Andre P. Kengne, Collet Dandara

**Affiliations:** 1 Division of Human Genetics, Department of Pathology, Faculty of Health Sciences, University of Cape Town, Cape Town, South Africa; 2 Institute of Infectious Disease and Molecular Medicine, University of Cape Town, Cape Town, South Africa; 3 Department of Medicine, Faculty of Health Sciences, University of Cape Town, Cape Town, South Africa; 4 Non-Communicable Diseases Research Unit, South African Medical research Council, Cape Town, South Africa

**Keywords:** Cytomegalovirus, Toll-like receptors, Interleukins, CMV DNA, Zimbabwe

## Abstract

**Background:**

Maternal cytomegalovirus (CMV) infection and/or reactivation in pregnancy is associated with a myriad of adverse infant outcomes. However, the role of host genetic polymorphisms in modulating maternal CMV status is inconclusive. This study investigated the possible association of single nucleotide polymorphisms in toll-like receptor (TLR) and cytokine genes with maternal plasma CMV DNA status in black Zimbabweans.

**Methods:**

In a cross-sectional study, 110 women in late gestation who included 36 CMV infected cases and 74 CMV uninfected, age and HIV status matched controls were enrolled. Twenty single nucleotide polymorphisms in 10 genes which code for proteins involved in immunity against CMV were genotyped using Iplex GOLD SNP genotyping protocol on the Agena MassARRAY® system. Statistical analyses were performed using Stata SE and the ‘Genetics’ and ‘SNPassoc’ packages of the statistical package R.

**Results:**

The *TLR7* rs179008A > T (*p* < 0.001) polymorphism was associated while the *TLR9* rs352139T > C (*p* = 0.049) polymorphism was on the borderline for association with CMV positive (CMV+) status. In contrast, the interleukin (*IL)-6* rs10499563T > C (*p* < 0.001) and *TLR2* rs1816702C > T (*p* = 0.001) polymorphisms were associated with CMV negative (CMV-) status. Furthermore, allele frequencies of SNPs in *TLR2, TLR4, TLR9, TLR7*, *IL*-*6*, *IL-10*, *IL-28B*, *IL-1A* and interferon AR1 (*IFNAR1*) genes are being reported here for the first time in a Zimbabwean population. The allele frequencies in the Zimbabwean population are generally comparable to other African populations but different when compared to European and Asian populations.

**Conclusions:**

Toll-like receptor and interleukin genetic polymorphisms influence CMV status in late gestation among black Zimbabweans. This is attributable to possible modulation of immune responses to CMV reactivation in a population previously exposed to CMV infection.

## Background

Seroprevalence of cytomegalovirus (CMV) amongst women of reproductive age ranges from 40 to 65% in the developed world and can reach 100% in developing countries [1, 2]. CMV infection in pregnancy, in the setting of both primary infection and reinfection, can be potentially transmitted to the foetus and or neonate, resulting in congenital CMV (cCMV). The consequences of CMV range from asymptomatic viraemia to potentially life changing conditions which include mental retardation and congenital sensorineural hearing loss. Studies have implicated maternal demographics, socioeconomics and HIV status among the strongest determinants of the biased occurrence and vertical transmission of CMV [3–7]. Furthermore, maternal immune responses to CMV infection and/or reactivation actively modulate CMV related disease outcomes [8]. Thus, variation in genes that encode components of the immune system that are directly or indirectly involved in the pathogenesis of CMV have been implicated in CMV infection outcomes [9]. However, the genetic variants, like seroprevalences and the factors influencing CMV epidemiology are heterogenous among populations hence research findings are equivocal.

Toll-like receptors (TLR) are crucial in the detection of viruses in circulation and the subsequent elicitation of an antiviral response [10, 11]. TLRs act as pattern recognition receptors of non-methylated viral CpG-containing DNA which signals the presence of CMV infection [12]. TLR2 and TLR4 are cell surface receptors while TLR3, − 7 and − 9 are endosomal receptors [13, 14]. TLRs facilitate viral attachment and entry resulting in CMV-elicited signalling antiviral responses such as type 1 interferon activation of nuclear factor kappa β (NF-k β) and pro-inflammatory cytokine gene expression [12, 15]. Activation of the type 1 interferon producing cascade and production of cytokines form the major cellular antiviral mechanisms against CMV [16–18]. Single nucleotide polymorphisms (SNPs) in the *TLR2, TLR4, TLR7* and *TLR9* genes were inconclusively reported to be associated with CMV infection [19–23].

In response to TLR activation, chemokine (interleukin and interferon) genes signal immediate secretion of ILs from cells such as macrophages and T-helper cells. Chemokines that trigger an immune cascade by signalling direct growth, development, maturation, activation and increased life-span of immune cells. In the case of CMV infection, chemokines signal: maturation of B-lymphocytes into plasma cells which produce anti-CMV antibodies, and activation of cytotoxic T cells for destruction of CMV infected cells [24, 25]. The differential response to CMV exposure with some but not all exposed individuals developing CMV-related diseases suggests a possible role of host genetic variation in immune response. A study by Sezgin et al. [26] showed that human interleukin-10 receptor variants potentially interfere with IL-10 binding and signal transduction influencing susceptibility to CMV retinitis. In a large Swiss HIV Cohort Study, the effect of *IFNL3* TT/−G substitution, the variant allele was associated with occurrence of CMV retinitis [27]. The same allele was also associated with susceptibility to CMV replication in transplant patients [28].

Detection of host genetic variants which may confer resistance to CMV infection and reactivation could reveal potential therapeutic targets against pregnancy related CMV disease. Furthermore, host genetic determinants of CMV disease outcomes could be used as predictors of adverse outcomes of maternal CMV. While the host genetics of CMV have been studied in other populations, a glaring gap in knowledge exists among Africans. The differences in genomic variation between Africans and other populations cannot be over-emphasised, hence findings from other populations may not be an accurate reflection in Africans.

The aim of the present study was to determine if single nucleotide polymorphisms in genes that encode components of the immune system are associated reactivation of CMV in late pregnancy.

## Methods

### Study participants

This study was carried out among pregnant women in late gestation, seeking antenatal care at three polyclinics in Harare’s Kuwadzana, Dzivarasekwa and Glenview high density suburbs who were recruited in the University of Zimbabwe College of Health Sciences Birth Cohort (MRCZ/A/1968). The general study design, setting and participants characteristics for the main cohort are described elsewhere [29]. In summary, this cross-sectional nested sub-study enrolled 110 women aged 18 to 42 years, including 36 CMV infected cases and 74 CMV uninfected, age- and HIV status matched controls. All participants previously tested positive for CMV IgG antibodies hence, cases were presumed to have reinfection/reactivation. Whole blood and plasma specimens archived at enrolment were retrieved for host genotyping and CMV DNA detection, respectively. CMV status of participants was determined by detection of CMV DNA in plasma using the real time polymerase chain reaction (PCR) kit (RealStar CMV Kit v1.0, Altona Diagnostics, Hamburg, Germany), following manufacturer’s instructions.

### Genotyping of candidate genes

Using candidate gene approach, 20 SNPs in 10 genes were selected for genotyping (Table 1). Selection of SNPS was based on the following criteria: previously reported association or plausible association with CMV infection and/or other viral infection, a minor allele frequency (MAF) ≥10% in African populations reported in the dbSNP database (Available from: http://www.ncbi.nlm.nih.gov/SNP/), except for the rs113181057 SNP whose MAF in African populations was not previously reported. Host genomic DNA was extracted from 200 μl of whole blood using the Quick-DNA™ MiniPrep Plus Kit (Zymo Research, Irvine, CA, USA), according to manufacturer’s instructions. All DNA samples were diluted to a concentration of approximately 50 ng/ul in preparation of genotyping. SNPs were genotyped using Iplex GOLD SNP genotyping protocol on the Agena MassARRAY® system (Agena BioscienceTM, San Diego, CA, USA).

**Table 1:** Single nucleotide polymorphisms included in this study

Gene	SNP	Chrom	Genomic region	Functional effect
*TLR2*	rs4696480T > A	4	Intron	↓transcriptional activity
*TLR2*	rs3804099C > T	4	Exon	↓protein activity
*TLR2*	rs1816702C > T	4	Intron	↑protein levels
*TLR4*	rs1554973C > T	9	3’UTR	↓transcriptional activity
*TLR4*	rs2737190G > A	9	5’UTR	↑transcriptional activity
*TLR4*	rs10759932T > C	9	Promoter	↑transcriptional activity
*TLR4*	rs7856729G > T	9	3’UTR	Not known
*TLR7*	rs179008A > T	X	Exon	↓protein activity
*TLR9*	rs352139T > C	3	Intron	↑transcriptional activity
*TLR9*	rs5743836A > G	3	Promoter	↓transcriptional activity
*TLR9*	rs187084A > G	3	Promoter	↓transcriptional activity
*TLR9*	rs352140C > T	3	Exon	Not known
*IL-6*	rs10499563T > C	7	Promoter	↑transcriptional activity
*IL-6R*	rs4537545T > C	1	Intron	Not known
*IL-10*	rs1800872G > T	1	Promoter	↑transcriptional activity
*IL-10*	rs1878672G > C	1	Intron	↑susceptibility to infection
*IL-28B*	rs12979860T > C	19	Intron	↓protein activity
*IFNAR1*	rs2843710C > G	21	5’UTR	↓transcriptional activity
*IFNAR1*	rs113181057T > C	21	Exon	↓protein activity
*IL-1A*	rs1800587T > C	2	5’UTR	↑transcriptional activity

Key: *SNP* Single nucleotide polymorphism, *Chrom* Chromosome number, *TLR* Toll-like receptor, *IL* Interleukin, *IFNAR* Interferon α, *UTR* Untranslated region, ↑ increased, ↓ decreased, *N/A* not reported, *NB* Functional effects accessed on dbSNP (http://www.ncbi.nlm.nih.gov/SNP/

### Statistical analysis

Data were compiled and managed in Research Electronic Data Capture (REDCap) [30]. Statistical analyses were performed using Stata SE, version 15 (StataCorp, College Station, Texas, USA) and the ‘Genetics’ and ‘SNPassoc’ packages of the statistical package R (version 3.4.3 [2017-11-30], The R Foundation for Statistical Computing, Vienna, Austria). Numerical variables are described as either median and 25th to 75th percentiles for skewed variables or mean and standard deviation for normally distributed variables, with groups comparisons via Mann-Witney U-test and Student’s t-test respectively. Categorical variables are described as frequencies and compared across groups using Chi squared test. *p*-value < 0.05 was considered statistically significant. Genotype and allele frequencies were calculated using ShesisPlus [31]. SNPs were tested for departure from Hardy-Weinberg Equilibrium (HWE) expectation using a Chi square goodness of fit test. Association between SNPs and CMV status was determined using univariable logistic regression analysis. Bonferroni correction was used to account for simultaneous comparison of multiple SNPs. Dominant, log-additive, codominant, recessive and overdominant inheritance models were interrogated for association of SNPs with CMV infection. Furthermore, multivariate logistic regression analysis of SNPs that were associated with CMV infection in the univariate analysis was carried out to adjust for their effect on each other in a model that also contained BMI as covariate.

## Results

### Study participants’ demographic and clinical characteristics

The demographic and clinical characteristics of the 110 participants are summarised in Table 2. All participants were of child bearing age (median 28 years, 25th–75th percentile: 23–34). The group of women with a positive CMV DNA (CMV+, *n* = 36) status (median 24 kg/m^2^, 25th–75th percentile: 22–27) had a significantly lower body max index (BMI) than the group who tested negative for CMV DNA (CMV-, *n* = 74) (median 26 kg/m^2^, 25th–75th percentile: 24–29); *p* = 0.006. CMV+ participants also had significantly lower systolic blood pressure when compared with the CMV- participants. Age, gestational age, parity, gravidity, diastolic blood pressure, pulse rate, income, level of education and HIV status were comparable between CMV+ cases and CMV- controls.

**Table 2:** Participants’ demographic and clinical characteristics

Characteristic	CMV- (*n* = 74)	CMV+ (*n* = 36)	*P*-value
^a^Age in years	29 (23–34)	28 (23–33)	0.85
^b^Gestational age, weeks	32.4 ± 4.8	32.1 ± 3.5	0.73
^b^SBP, mmHg ± sd	113 ± 14	109 ± 9	0.037
^b^DBP, mmHg ± sd	70 ± 10	67 ± 9	0.13
^b^Pulse rate, bpm ± sd	82 ± 10	80 ± 12	0.33
^a^BMI	26.3 (24.3–28.8)	24.2 (21.7–27.3)	0.006
^a^parity	1 (0–2)	1 (0–2)	0.31
^a^gravidity	3 (2–4)	2 (1–3)	0.62
HIV infected n (%)	45 (61)	28 (78)	0.08
^a^income in USD/month	235 (171–300)	225 (153–332)	0.97
Education n (%)			0.30
Secondary	67 (91)	30 (83)	
Primary	4 (5)	5 (14)	
Tertiary	3 (4)	1 (3)	

Key: *CMV* Cytomegalovirus, *CMV +* CMV infected, *CMV-* CMV uninfected, *BMI* Body mass index, ^a^given as median and interquartile range, ^b^given as mean and standard deviation

### Association between SNPs and CMV infection

Genotype data for the 20 SNPs genotyped was available for all 110 participants and the SNP rs113181057 on the *IFNAR1* gene was monomorphic in the study population. There was a departure from Hardy-Weinberg equilibrium (HWE) for four of the 20 SNPs: *TLR7* rs179008 in cases, *TLR2* rs1816702 and *IL-6* rs10499563 in the controls and *IFNAR1* rs2843710 in both groups. An additional table shows genotype frequencies in the CMV+ and CMV- negative groups and the univariate logistic regression analyses of SNPs and CMV status (see Additional Table 1). Using the univariate logistic regression analysis of codominant and log additive inheritance models, 4 SNPs (rs10499563 (*p* < 0.001), rs179008 (*p* < 0.001), rs1816702 (*p* = 0.002) and rs352139 (*p* = 0.003) were significantly associated with CMV DNA status (Additional Table 1). The *IL-6* rs10499563T > C polymorphism was significantly associated with lower risk of CMV infection. When compared to the *IL-6* rs10499563T/T genotype, the rs10499563T/C was associated with a lower risk of CMV infection as the genotype was significantly (*p* < 0.001) less frequent in the CMV+ group (14%) than the CMV- group (70%). Likewise, the *TLR2* rs1816702C > T SNP was significantly associated with lower risk of CMV infection. Genotype rs1816702C/C genotype was significantly (*p* = 0.002) higher in the CMV+ (47%) than the CMV- women (11%).

**Fig. 1:**
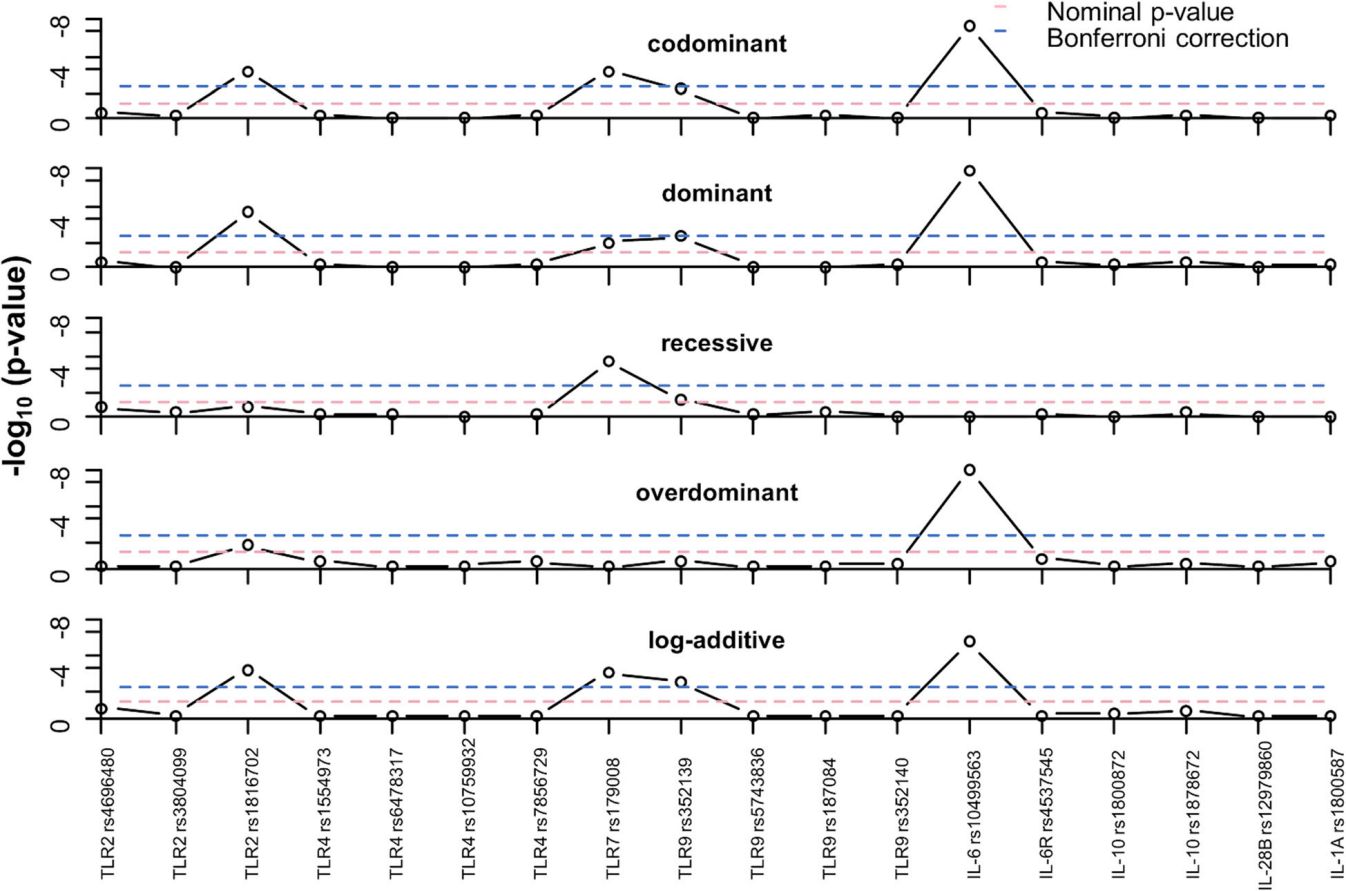
Plot of log_10_*p*-values for the association of gene with CMV DNA across models of genetic associations. For each figure panel, the lower dotted horizontal line is for the nominal *p*-value threshold for significance (0.05), while the upper dotted blue line for the Bonferroni corrected threshold *p*-value for significance

In contrast, *TLR7* (rs179008A > T) and *TLR9* (rs352139T > C) polymorphisms were associated with an increased risk of CMV infection. The *TLR7* rs179008C/C genotype was significantly higher in the CMV+ group than the CMV- group (31% vs. 3%; *p* < 0.001. With reference to the *TLR9* rs352139T/T genotype, both the rs352139T/C and rs352139C/C genotypes were significantly (*p* = 0.005) higher in the CMV+ women (28 and 58% respectively) than in the CMV- women (11 and 47% respectively). These associations remained significant after correction for multiple comparisons (Fig. 1). When other models of genetic inheritance were considered, the association of *IL-6* rs10499563 maintained significant association with CMV status after Bonferonni correction (BC) in dominant, and overdominant models. SNPs rs1816702 and rs179008 also maintained significance with CMV status after BC in the dominant and recessive models (Fig. 1).

Table 3 shows multivariable logistic regression analysis of SNPs (rs10499563, rs179008, rs1816702 and rs352139) that were associated with CMV status in the univariable analyses. BMI was also included in the model. All SNPs maintained significant association with CMV infection status in at least one of the models. However significant association of rs352139 with CMV status was borderline (*p* = 0.049) in the log additive model while it was not significant in the other models. BMI’s association with CMV status also substantially attenuated in the multivariable logistic regression model (*p* = 0.068).

**Table 3:** Multivariable adjusted models containing BMI and significant SNPs in univariable analysis for CMV status

SNP	Model	Genotype	OR (95%CI)	*p*-value
*TLR2* rs1816702	Codominant	C/T	0.09 (0.02–0.43)	0.001
		T/T	0.06 (0.01–0.48)	
	Dominant	C/T-T/T	0.08 (0.02–0.37)	0.0003
	Recessive	T/T	0.32 (90.07–1.50)	0.133
	Overdominant	C/T	0.29 (0.08–1.01)	0.044
	Log additive	0,1,2	0.22 (0.08–0.62)	0.001
*TLR7* rs179008	Codominant	A/T	3.67 (0.79–116.99)	0.011
		T/T	18.69 (1.59–220.04)	
	Dominant	A/T-T/T	6.05 (1.53–23.94)	0.006
	Recessive	T/T	13.15 (1.15–149.74)	0.013
	Overdominant	A/T	2.27 (0.53–9.68)	0.262
	Log additive	0,1,2	4.08 (1.46–11.39)	0.003
*TLR9* rs352139	Codominant	T/C	2.87 (0.50–16.58)	0.144
		C/C	8.13 (0.90–73.63)	
	Dominant	T/C-C/C	3.58 (0.65–19.66)	0.121
	Recessive	C/C	3.65 (0.67–19.77)	0.124
	Overdominant	T/C	1.05 (0.30–3.68)	0.938
	Log additive	0,1,2	2.85 (0.95–8.58)	0.049
*Il-6* rs10499563	Codominant	T/C	0.05 (0.01–0.25)	< 0.001
		C/C	0.42 (0.05–3.77)	
	Dominant	T/C-C/C	0.08 (0.02–0.31)	< 0.001
	Recessive	C/C	1.53 (0.21–11.39)	0.682
	Overdominant	T/C	0.06 (0.01–0.27)	< 0.001
	Log additive	0,1,2	0.19 (0.06–0.58)	0.001

The *IL-6* rs10499563T > C SNP was associated with low likelihood of CMV positivity in codominant (OR = 0.05; 95%CI = 0.01–0.25, *p* = 0.001) as well as in the log additive, dominant and overdominant models. The result shows the association of the C allele with lower odds of CMV infection even in heterozygous state (rs10499563 T/C). For the *TLR7* rs179008A > T SNP, the T allele was significantly associated with higher odds of CMV infection in the codominant model (OR = 3.67; 95%CI = 0.79–116.99; *p* < 0.011) which, was maintained in the log additive, recessive and dominant models. Hence T allele will likely be associated with CMV+ status in both homozygous and heterozygous states (rs179008T/T and rs179008A/T). The *TLR2* rs1816702T > C was significantly associated with decreased risk of CMV positivity both in the codominant (OR = 0.05; 95%CI = 0.01–0.25, *p* = 0.001) as well as in the log additive, dominant and overdominant models. Hence, risk of CMV infection will be decreased in the homozygous state, rs1816702C/C. The TLR9 rs352139 was significantly associated with likelihood of CMV infection, only in the log additive model (OR = 2.85: 95%CI = 0.95–8.58; *p* = 0.049).

### Comparison of variant allele frequencies from this study with other populations

The variant allele frequencies of the genotyped SNPs were compared with data from two other populations: Asians and Europeans. Table 4 gives variant allele frequencies for the genotyped SNPs in this study as well as for Asians and Europeans as reported on dbSNP.

**Table 4:** Comparison of variant allele frequencies of genotyped SNPs with other populations

Gene	SNP	Variant allele	Zimbabwean (This study)	Other Africans	Europeans	Asians
*TLR2*	rs4696480	A	0.31	0.37	0.52	0.57
*TLR2*	rs3804099	T	0.45	0.36	0.56	0.72
*TLR2*	rs1816702	T	0.47	0.43	0.12	0.00
*TLR4*	rs1554973	T	0.20	0.21	0.77	0.86
*TLR4*	rs2737190	A	0.14	0.16	0.33	0.37
*TLR4*	rs10759932	C	0.19	0.25	0.85	0.76
*TLR4*	rs7856729	T	0.38	0.33	0.13	0.10
*TLR7*	rs179008	T	0.23	0.12	0.23	0.00
*TLR9*	rs352139	C	0.42	0.61	0.55	0.40
*TLR9*	rs5743836	G	0.36	0.42	0.13	0.00
*TLR9*	rs187084	G	0.30	0.29	0.43	0.40
*TLR9*	rs352140	T	0.32	0.29	0.55	0.39
*IL-6*	rs10499563	C	0.31	0.27	0.23	0.16
*IL-6R*	rs4537545	C	0.26	0.34	0.37	0.32
*IL-10*	rs1800872	T	0.40	0.44	0.24	0.68
*IL-10*	rs1878672	C	0.25	0.26	0.45	0.05
*IL-28B*	rs12979860	G	0.70	0.82	0.86	0.97
*IFNAR1*	rs2843710	G	0.30	0.31	0.41	0.36
*IFNAR1*	rs113181057	C	0.00	N/A	N/A	N/A
*IL-1A*	rs1800587	C	0.65	0.60	0.71	0.93

Key: *SNP* Single nucleotide polymorphism, *TLR* Toll-like receptor, *IL* Interleukin, *IFNAR* Interferon α

## Discussion

The outcome of an infection is determined, in part, by the intensity of the inflammatory response [32], which varies between individuals and can be regulated at the genetic level [33]. In this study, we hypothesised the possible contribution of genetic variation to the biased occurrence of CMV infection among pregnant women. SNPs may influence the rate and regulatory dynamics of gene transcription, stability of mRNA as well as production and biological activity of resultant protein. We therefore investigated possible association between CMV infection and SNPs in 19 genes which encode proteins that are or may be involved in the immune reaction cascade against CMV. The departure from HWE in polymorphic SNPs is due to their association with CMV infection mainly because the departure is being observed when cases and controls are separated but HWE is maintained when the two groups are combined. We report a significant association between each of; rs10499563, rs179008, rs1816702 and rs352139 SNPs and CMV DNA status. To our knowledge, this is the first report on SNPs and CMV infection in an African setting.

To minimise the confounding effects of age and HIV status, which are directly related to immune function, enrolled participants were age and HIV status matched. The observation that overweight women were less likely to be CMV+ contradicts findings from previous studies where CMV infection was associated with metabolic syndrome, higher BMI and or obesity [34, 35]. Our findings could be due to none of the participants having any form or history of metabolic syndrome. Hence, we were unlikely to observe any significant associations. The observation that CMV positivity is significantly associated with low systolic blood pressure contrasts with previous findings which have shown increasing systolic blood pressure with CMV positivity [36, 37]. It is worth noting that the previous studies were carried out in non-pregnant adults, hence discrepancy in findings could be due to the well documented effects of pregnancy on fluctuations in blood pressure [38, 39] masking the effects of CMV infection.

We found an association between SNP rs10499563 (− 6331 T > C), located within the promoter region of *IL-6* gene which regulates the rate of IL-6 gene transcription [40] and CMV DNA status. Individuals carrying the C allele were less likely to be CMV infected, hence likelihood of being CMV DNA positive decreased with genotypes T/T>> > T/C> > C/C. Individuals heterozygous (T/C) and homozygous (C/C) for the variant allele were significantly less likely to be CMV infected than individuals homozygous for the T allele (T/T). The *IL-6* gene codes for IL-6, a versatile inflammatory cytokine whose function is related to its expression in the tissue. Smith et al. previously reported higher level of serum IL-6, in individuals with wildtype T/T genotype compared to individuals with C/C genotype, among coronary artery bypass patients (Smith et al. [41]).

Our findings could at least in part, be explained by results from the Smith et al. study. Being a pro-inflammatory cytokine, abundance of IL-6 in circulation could promote CMV activation. In contrast, the low levels of IL-6 associated with the rs10499563C allele would disfavour the occurrence of CMV infection. Serum IL-6 levels were reported to be significantly higher among the CMV infected pregnant women compared to the CMV uninfected in a Chinese cohort [42].

We also report an association between CMV DNA status and rs179008, a non-synonymous A > T (Gln11Leu) polymorphism within exon 3 of the *TLR7* gene [43]. The resulting glycine to leucine change has been suggested to code for a functionally impaired TLR7 protein [44, 45]. In the present study, the T allele was associated with significantly lower odds of CMV positivity. Individuals homozygous for the variant allele T/T were significantly less likely to be CMV infected compared to individuals homozygous for the wildtype allele A/A.

Upon recognising pathogen associated molecular patterns (PAMP), TLR7 activate a signalling cascade which activates type I IFN, dendritic cells (DCs) and B lymphocytes [46]. Activated type 1 IFN, DCs and B cells are responsible for pathogen clearance, antigen recognition and antibody production. The induced immune cascade is critical in CMV clearance. In the presence of the T allele which results in a less potent protein, an insufficient signal is mounted by TLR7, hence carriers of the rs179008 T allele are at a greater risk of CMV infection. The rs179008 T allele has been linked with unfavourable outcomes in HIV and other viral infections. The variant was associated with increased susceptibility to HIV-1 and decreased IFNα production in HIV uninfected women [47]. The T allele has also been previously associated with a higher risk of hepatitis C infection and cCMV. Our findings are therefore contrasting with previous reports suggesting that the rs179008A > T SNP could be in linkage disequilibrium with another functional SNP or epistatic gene which masks the effects of rs179008A > T.

CMV DNA status was also associated with rs1816702C > T, a SNP located in intron 2 of the *TLR2* gene. The C variant was significantly more prevalent in cases than in controls which means that participants with the rs1816702 C/C genotype were at a higher risk of being CMV+ than those with rs1816702 T/T genotype. TLR2 recognise CMV glycoproteins B (gB) and gH in a process which facilitates entry of CMV into immune cells [15, 48]. The rs1816702T allele is associated with significantly elevated levels of inflammatory monocytes expressing CD14+/TLR2+ receptors than rs1816702C allele [49]. This could explain our findings of a higher risk of CMV among rs1816702C/C carriers because their immune response against CMV is impaired due to lower TLR2 expression compared to the T/T. Homozygosity for the rs1816702C allele has also been associated with increased odds of *Mycobacteria leprae* infection and inflammatory bowel disease which were attributable to altered NFκB-mediated inflammatory response [50, 51].

The intronic SNP rs352139T > C in the *TLR9* gene was also associated with CMV DNA status. Homozygous rs352139C/C individuals were at a significantly higher risk of being CMV+ compared to homozygous T/T carriers. The effect of the C allele on risk of CMV infection was also observed in the dominant and recessive models where the significance of the compound heterozygous (T/C) and homozygous (C/C) genotypes had a greater risk than the homozygous (C/C) alone, relative to the T/T genotype in both cases. The higher risk of CMV positivity in homozygous carriers of the C allele suggest that the polymorphism results in a less potent protein compared to the T allele. Since the polymorphism is intronic, it likely creates an alternative splicing site thus, affecting mRNA transcription and the final protein product. A less potent protein would have decreased ability to form dimers that are required to illicit an immune reaction. Individuals who are homozygous T/T have impaired immune responses against CMV infection, hence are more likely to experience CMV infection or reactivation. The HIV rapid progressor phenotype has been linked to homozygosity for rs352139T allele also due to reduced TRL9 potency [52].

Conflicting findings were reported reduced risk of cCMV associated with the rs352139T/T genotype among infants in Poland [53]. The conflicting effect of rs352139T variant have also been reported in bacterial infection studies in Indonesia and Mexico, perhaps due to ethnic differences [54, 55]. We suggest that rs352139 could be in linkage disequilibrium (LD) with a polymorphic regulatory region that controls *TLR9* expression or serves as a functional region SNP. LD patterns differ with level of genetic diversity among different ethnic groups, hence the effects of one SNP may vary from one population to another. Minor allele frequencies for these SNPs which seem to affect CMV infection risk were compared to other populations. TLR2 rs4696480A and TLR4 rs1075993T alleles, respectively, have lower frequencies among Zimbabweans (0.31 and 0.20) and other African populations (0.37 and 0.21) when compared to European (0.52 and 0.77) and Asian (0.57 and 0.86) populations. On the other hand, TLR4 rs7856729T and TLR9 rs5743836G, respectively, are proportionally higher in Zimbabwean (0.38 and 0.36) and other Africans (0.33 and 0.42) when compared to European (0.13 and 0.13) and Asian (0.10 and 0.00) populations. These differences in the distribution of risk alleles of world populations, is likely to lead to differential responses upon exposures to infectious pathogens. Indeed, the adaptive immune responses to the β-coronaviruses, MERS-CoV and SARS-CoV, are that can cause fatal lower respiratory tract infections, are marshalled by T cells, CD4+ T cells, and CD8+ T cells, through among other processes, activate other downstream cytokine and chemokine cascades, such as IL-1, IL-6, IL-8, IL-21 and TNF-β [56]. The molecular patterns displayed by viruses are then sensed by different immune cellular pathogen recognition receptors, including toll-like receptors (TLR:2, 3, 4, 7, 8, and 9) [57]. Whether this genetic heterogeneity among populations plays an active role in the differential prevalence of CMV is unclear and is an area of further research which should also consider the strong influence of environmental factors.

## Conclusions

We conclude that *TLR2, − 7, − 9* and *IL-6* genetic polymorphisms are associated CMV status in late gestation among the black Zimbabweans. TLRs and ILs modulate immune responses to CMV, hence polymorphisms in genes encoding the receptors and cytokines could interfere with the immune mechanisms, hence their association with CMV status. We recommend that future studies consider evaluating the profiles of immune response genes and the polymorphisms in these genes on their possible effects in viral infections. With respect to CMV, we recommend a mother-infant longitudinal approach that will seek to factor in the effect of these immunogenetic profiles in congenital CMV and its possible sequelae.

## Supplementary information

**Additional file 1: Table 1.** Genotype frequencies and univariate logistic regression of SNP with CMV infection status.

## Data Availability

The datasets used and/or analysed during the current study are available from the corresponding author on request or in the dbSNP repository, http://www.ncbi.nlm.nih.gov/SNP/snp_viewTable.cgi?handle=HUMGEN_PHARMGX

## Abbreviations

CMV: Cytomegalovirus
TRL: Toll-like receptor
SNP: Single nucleotide polymorphism
IL: Interleukin
IFN: Interferon
HWE: Hardy-Weinberg Equilibrium
BMI: Basal metabolic index
PAMP: Pathogen associated molecular pattern
DC: Dendritic cell
NFκB: Nuclear factor kappa β
