# Supplementary material for: Genetic variation in toll like receptors 2, 7, 9 and interleukin-6 is associated with cytomegalovirus infection in late pregnancy
Source: BMC Med Genet. 2020 May 25;21:113. doi: 10.1186/s12881-020-01044-8 (PMC7247288; doi:10.1186/s12881-020-01044-8)
Supplement: Supplementary file 1 — Additional file 1: Table 1. Genotype frequencies and univariate logistic regression of SNP with CMV infection status. [file 12881_2020_1044_MOESM1_ESM.docx]

**Additional Table 1** Genotype frequencies and univariate logistic regression of SNP with CMV infection status

| **SNP** | **Genotype** | **overall** | **CMV+ n (%)** | **CMV- n (%)** | **p-value** | **Codominant OR (95%CI)** | **p-value** | **Log additive OR** | **p-value** |
| --- | --- | --- | --- | --- | --- | --- | --- | --- | --- |
| *TLR2* rs4696480 | T/T | 50 (45.4) | 14 (38.9) | 36 (48.6) | 0.295 | 1.00 | 0.315 |  |  |
|  | T/A | 53 (48.2) | 18 (50.0) | 35 (47.3) |  | 1.32 (0.57-3.06) |  |  |  |
|  | A/A | 7 (6.4) | 4 (11.1) | 3 (4.0) |  | 3.43 (0.68-17.31) |  | 1.58 (0.82-3.06) | 0.172 |
| *TLR2* rs3804099 | C/C | 54 (49.1) | 18 (50.0) | 36 (48.6) | 0.688 | 1.00 | 0.658 |  |  |
|  | C/T | 50 (45.4) | 17 (47.2) | 33 (44.6) |  | 1.03 (0.46-2.33) |  |  |  |
|  | T/T | 6 (5.4) | 1 (2.8) | 5 (6.8) |  | 0.40 (0.04-3.68) |  | 0.86 (0.44-1.69) | 0.659 |
| *TLR2* rs1816702 | C/C | 25 (22.7) | 17 (47.2) | 8 (10.8) | <0.001 | 1.00 | <0.001 |  |  |
|  | C/T | 64 (58.2) | 15 (41.7) | 49 (66.2) |  | 0.14 (0.05-0.40) |  |  |  |
|  | T/T | 21 (19.1) | 4 (11.1) | 17 (23.0) |  | 0.11 (0.03-0.44) |  | 0.27 (0.13-0.57) | <0.001 |
| *TLR4* rs1554973 | C/C | 72 (65.4) | 25 (69.4) | 47 (63.5) | 0.568 | 1.00 | 0.564 |  |  |
|  | C/T | 31 (28.2) | 8 (22.2) | 23 (31.1) |  | 0.65 (0.26-1.67) |  |  |  |
|  | T/T | 7 (6.4) | 3 (8.3) | 4 (5.4) |  | 1.41 (0.29-6.80) |  | 0.92 (0.47-1.79) | 0.807 |
| *TLR4* rs6478317 | G/G | 82 (74.5) | 27 (75.0) | 55 (74.3) | 0.853 | 1.00 | 0.860 |  |  |
|  | G/A | 26 (23.6) | 8 (22.2) | 18 (24.3) |  | 0.91 (0.35-2.34) |  |  |  |
|  | A/A | 2 (1.8) | 1 (2.8) | 1 (1.3) |  | 2.04 (0.12-33.83) |  | 1.03 (0.46-2.34) | 0.939 |
| *TLR4* rs10759932 | T/T | 69 (62.7) | 22 (61.1) | 47 (63.5) | 0.740 | 1.00 | 0.888 |  |  |
|  | T/C | 40 (36.4) | 14 (38.9) | 26 (35.1) |  | 1.15 (0.50-2.62) |  |  |  |
|  | C/C | 1 (0.9) | 0 | 1 (1.3) |  | - |  | 1.04 (0.47-2.29) | 0.888 |
| *TLR4* rs7856729 | G/G | 41 (37.3) | 15 (41.7) | 26 (35.1) | 0.633 | 1.00 | 0.632 |  |  |
|  | G/T | 53 (48.2) | 15 (41.7) | 38 (51.3) |  | 0.68 (0.29-1.64) |  |  |  |
|  | T/T | 16 (14.5) | 6 (16.7) | 10 (13.5) |  | 1.04 (0.31-3.44) |  | 0.93 (0.52-1.67) | 0.807 |
| *TLR7* rs179008 | A/A | 71 (64.5) | 17 (47.2) | 54 (73.0) | <0.001 | 1.00 | <0.001 |  |  |
|  | A/T | 26 (23.6) | 8 (22.2) | 18 (24.3) |  | 1.41 (0.52-3.82) |  |  |  |
|  | T/T | 13 (11.8) | 11 (30.6) | 2 (2.7) |  | 17.47 (3.52-86.72) |  | 2.97 (1.63-5.43) | <0.001 |
|  | HWE (p-value) | 0.0009 | 0.001 | 0.652 |  |  |  |  |  |
| *TLR9* rs352139 | T/T | 34 (32.1) | 5 (13.9) | 29 (41.4) | 0.007 | 1.00 | 0.005 |  |  |
|  | T/C | 54 (50.9) | 21 (58.3) | 33 (47.1) |  | 3.69 (1.23-11.04) |  |  |  |
|  | C/C | 18 (17.0) | 10 (27.8) | 8 (11.4) |  | 7.25 (1.92-27.37) |  | 2.70 (1.41-5.17) | 0.001 |
| *TLR9* rs5743836 | A/A | 43 (40.6) | 13 (39.4) | 30 (41.1) | 0.830 | 1.00 | 0.835 |  |  |
|  | A/G | 50 (47.2) | 15 (45.5) | 35 (47.9) |  | 0.99 (0.41-2.40) |  |  |  |
|  | G/G | 13 (12.3) | 5 (15.1) | 8 (11.0) |  | 1.44 (0.40-5.26) |  | 1.14 (0.62-2.10) | 0.675 |
| *TLR9* rs187084 | A/A | 54 (49.5) | 18 (50.0) | 36 (49.3) | 0.638 | 1.00 | 0.616 |  |  |
|  | A/G | 45 (41.3) | 16 (44.4) | 29 (39.7) |  | 1.10 (0.48-2.54) |  |  |  |
|  | G/G | 10 (9.2) | 2 (5.6) | 8 (11.0) |  | 0.50 (0.10-2.60) |  | 0.86 (0.46-1.61) | 0.645 |
| *TLR9* rs352140 | C/C | 48 (43.6) | 17 (47.2) | 31 (41.9) | 0.788 | 1.00 | 0.788 |  |  |
|  | C/T | 54 (49.1) | 16 (44.4) | 38 (51.3) |  | 0.77 (0.33-1.76) |  |  |  |
|  | T/T | 8 (7.3) | 3 (8.3) | 5 (6.8) |  | 1.09 (0.23-5.15) |  | 0.90 (0.47-1.74) | 0.763 |
| *IL-6* rs10499563 | T/T | 47 (43.1) | 29 (80.6) | 18 (24.7) | <0.001 | 1.00 | <0.001 |  |  |
|  | T/C | 56 (51.4) | 5 (13.9) | 51 (69.9) |  | 0.06 (0.02-0.18) |  |  |  |
|  | C/C | 6 (5.5) | 2 (5.6) | 4 (5.5) |  | 0.31 (0.05-1.87) |  | 0.13 (0.06-0.33) | <0.001 |
| *IL-6R* rs4537545 | T/T | 63 (57.3) | 23 (63.9) | 40 (54.0) | 0.399 | 1.00 | 0.390 |  |  |
|  | T/C | 37 (33.6) | 9 (25.0) | 28 (37.8) |  | 0.56 (0.23-1.39) |  |  |  |
|  | C/C | 10 (9.1) | 4 (11.1) | 6 (8.1) |  | 1.16 (0.30-4.54) |  | 0.85 (0.46-1.58) | 0.607 |
| *IL-10* rs1800872 | G/G | 38 (34.5) | 11 (30.6) | 27 (36.5) | 0.825 | 1.00 | 0.823 |  |  |
|  | G/T | 58 (52.7) | 20 (55.6) | 38 (51.3) |  | 1.29 (0.53-3.13) |  |  |  |
|  | T/T | 14 (12.7) | 5 (13.9) | 9 (12.2) |  | 1.36 (0.37-5.00) |  | 1.20 (0.65-2.20) | 0.564 |
| *IL-10* rs1878672 | G/G | 60 (54.5) | 22 (61.1) | 38 (51.3) | 0.515 | 1.00 | 0.494 |  |  |
|  | G/C | 44 (40.0) | 13 (36.1) | 31 (41.9) |  | 0.72 (0.31-1.67) |  |  |  |
|  | C/C | 6 (5.4) | 1 (2.8) | 5 (6.8) |  | 0.35 (0.04-3.15) |  | 0.67 (0.33-1.35) | 0.253 |
| *IL-28B* rs12979860 | T/T | 46 (47.9) | 16 (50.0) | 30 (46.9) | 0.948 | 1.00 | 0.948 |  |  |
|  | T/C | 32 (33.3) | 10 (31.2) | 22 (34.4) |  | 0.85 (0.33-2.23) |  |  |  |
|  | C/C | 18 (18.7) | 6 (18.7) | 12 (18.7) |  | 0.94 (0.30-2.97) |  | 0.95 (0.54-1.66) | 0.850 |
| *IFNAR1* rs2843710 | C/C | 8 (15.1) | 1 (6.7) | 7 (18.4) | 0.282 | 1.00 | 0.250 |  |  |
|  | C/G | 45 (84.9) | 14 (93.3) | 31 (81.6) |  | 3.16 (0.35-28.20) |  | 3.16 (0.35-28.20) | 0.250 |
|  | G/G | 0 | 0 | 0 |  | - |  |  |  |
| *IL-1A* rs1800587 | T/T | 46 (45.1) | 17 (50.0) | 29 (42.6) | 0.692 | 1.00 | 0.690 |  |  |
|  | T/C | 42 (41.2) | 12 (35.3) | 30 (44.1) |  | 0.68 (0.28-1.68) |  |  |  |
|  | C/C | 14 (13.7) | 5 (14.7) | 9 (13.2) |  | 0.95 (0.27-3.30) |  | 0.89 (0.49-1.60) | 0.688 |
